# IGF-I induces senescence of hepatic stellate cells and limits fibrosis in a p53-dependent manner

**DOI:** 10.1038/srep34605

**Published:** 2016-10-10

**Authors:** Hitoshi Nishizawa, Genzo Iguchi, Hidenori Fukuoka, Michiko Takahashi, Kentaro Suda, Hironori Bando, Ryusaku Matsumoto, Kenichi Yoshida, Yukiko Odake, Wataru Ogawa, Yutaka Takahashi

**Affiliations:** 1 Division of Diabetes and Endocrinology, Kobe University Hospital, Japan; 2 Department of Nutrition, Kobe University Hospital, Japan; 3 Division of Diabetes and Endocrinology, Department of Internal Medicine, Kobe University Graduate School of Medicine, Kobe, Japan

## Abstract

Hepatic fibrosis in nonalcoholic steatohepatitis (NASH) and cirrhosis determines patient prognosis; however, effective treatment for fibrosis has not been established. Oxidative stress and inflammation activate hepatic stellate cells (HSCs) and promote fibrosis. In contrast, cellular senescence inhibits HSCs’ activity and limits fibrosis. The aim of this study was to explore the effect of IGF-I on NASH and cirrhotic models and to clarify the underlying mechanisms. We demonstrate that IGF-I significantly ameliorated steatosis, inflammation, and fibrosis in a NASH model, methionine-choline-deficient diet-fed db/db mice and ameliorated fibrosis in cirrhotic model, dimethylnitrosamine-treated mice. As the underlying mechanisms, IGF-I improved oxidative stress and mitochondrial function in the liver. In addition, IGF-I receptor was strongly expressed in HSCs and IGF-I induced cellular senescence in HSCs *in vitro* and *in vivo*. Furthermore, in mice lacking the key senescence regulator p53, IGF-I did not induce cellular senescence in HSCs or show any effects on fibrosis. Taken together, these results indicate that IGF-I induces senescence of HSCs, inactivates these cells and limits fibrosis in a p53-dependent manner and that IGF-I may be applied to treat NASH and cirrhosis.

NASH is a common liver disease that is characterized histologically by hepatic steatosis, lobular inflammation, and hepatocellular ballooning in the absence of a history of significant alcohol use or other known liver disease^1^. The prevalence of NASH has been increasing rapidly world-wide^2^. NASH is a serious disease because it progresses to liver cirrhosis and hepatocellular carcinoma, and a specific treatment has not been established, particularly for the fibrosis^2^. Furthermore, liver cirrhosis is caused by not only by NASH but also by viral hepatitis, autoimmune hepatitis, and primary biliary cirrhosis and the degree of fibrosis determines prognosis, development of efficacious treatment for fibrosis is crucial.

The progression of NASH has been explained using the ‘two-hit’ model^3^. The first hit is the development of fatty liver in the context of obesity and consequent insulin resistance. The second hit encompasses the interplay between oxidative stress and inflammation, resulting in the activation of hepatic stellate cells (HSCs), which are key players in fibrosis^3^. HSCs receive a wide range of signals from injured hepatocytes and the perturbed hepatic microenvironment, most of which are mediated by cytokines. HSCs are highly responsive to pro-inflammatory cytokines and lipopolysaccharide (LPS), resulting in the activation, proliferation, and production of extracellular matrix, which is a hallmark of the fibrotic scar. Although the inhibitory mechanisms of HSCs are largely unknown, it has been reported that cellular senescence of activated HSCs limits liver fibrosis^4^. In mice lacking the key senescence regulator p53, cellular senescence in HSCs was not induced, leading to excessive liver fibrosis; however, the regulator of senescence remains unknown.

Growth hormone (GH) plays an essential role in adults as in adult patients with GH deficiency, visceral adiposity, abnormal lipid profile, insulin resistance, and increased prevalence of non-alcoholic fatty liver disease (NAFLD) are observed^5^,^6^,^7^. GH induces insulin-like growth factor-I (IGF-I) production mainly in the liver and regulates metabolism through its direct and indirect actions via IGF-I^7^,^8^. GH-deficient rats exhibited NASH and administration of GH or IGF-I restored these liver damages, indicating that IGF-I plays an important role in the liver^9^. Interestingly, oxidative stress was enhanced and mitochondrial function was impaired in the rats and administration of GH or IGF-I ameliorated these changes. In humans, patients with adult GH deficiency showed an increased prevalence of NAFLD/NASH and GH replacement therapy improved the liver both biochemically and histologically^5^,^10^. Furthermore, serum IGF-I levels were found to be decreased in patients with NAFLD^11^ and decreased IGF-I levels were associated with inflammation and fibrosis^12^,^13^, suggesting that a decrease in serum IGF-I levels may play a role in the development of NAFLD/NASH in a general population.

It has been suggested that IGF-I may be beneficial in the treatment of liver cirrhosis^14^. Systemic administration of IGF-I improved nutritional status and liver function, and improved fibrosis in a Carbon tetrachloride (CCl_4_)-induced cirrhotic animal model^15^,^16^,^17^,^18^. It has also been reported that forced expression or administration of IGF-I in a cirrhotic animal model increased food intake^19^, reduced portal pressure, improved endotoxemia, bacterial translocation^20^, and fibrosis^15^,^20^,^21^. These effects were associated with the antioxidant, antiapoptotic, antifibrogenic, and mitochondrial-protective effects of IGF-I^22^,^23^, although the precise mechanisms remain unclear. Here, we investigated the effect of IGF-I on NASH and cirrhotic animal models and demonstrate IGF-I ameliorated steatosis, inflammation, and fibrosis. In addition, we show a novel action of IGF-I on HSCs as the underlying mechanisms.

## Results

### IGF-I administration ameliorated NASH

**Figure 1:**
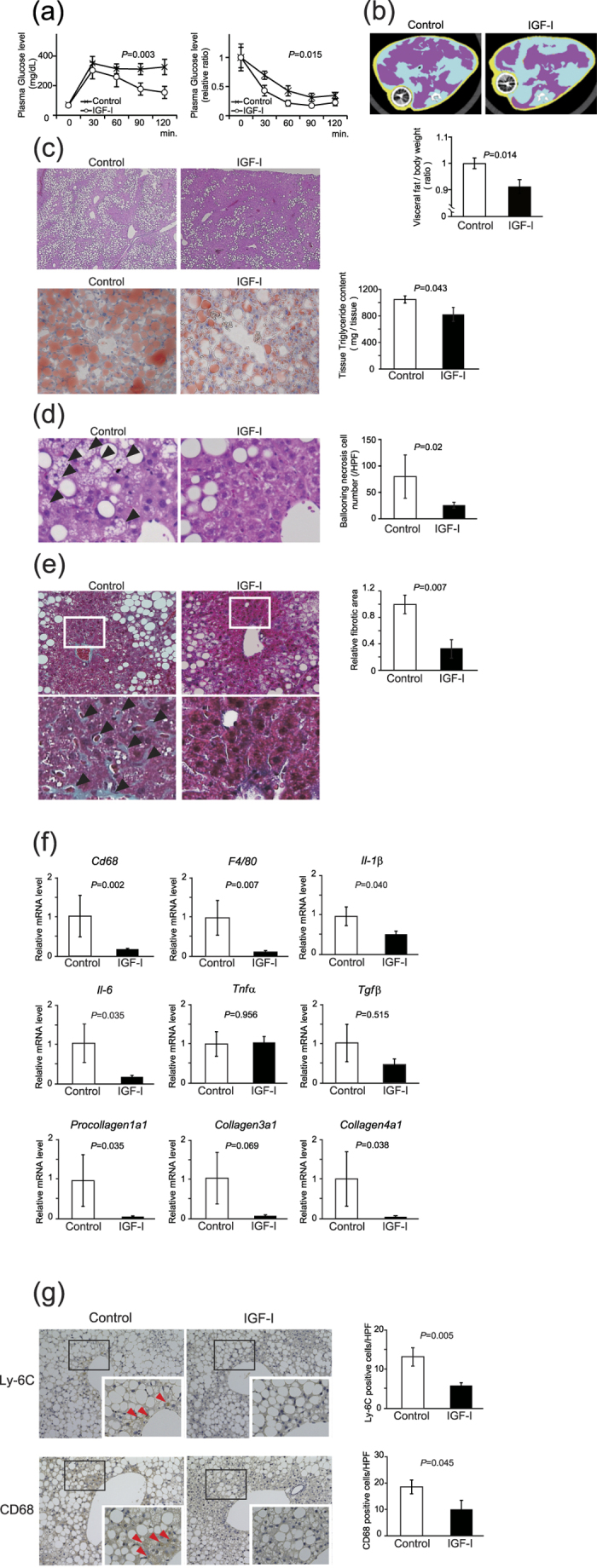
IGF-I improved glucose tolerance, visceral fat area, hepatocyte injury, and fibrosis in NASH model mice. MCD-*db/db* mice were treated with vehicle or IGF-I for 4 weeks using osmotic pump (n = 5 for each group). (**a**) Intraperitoneal glucose tolerance test and intraperitoneal insulin tolerance test. Data were compared by MANOVA test. (**b**) Visceral fat area visualized by computed tomography. Red areas show intraperitoneal fat. The osmotic infusion pump revealed a round-shaped and darkened figure. Quantitative analysis shows decreased visceral fat area in the IGF-I treatment group. (**c**) Histological analysis of the liver (hematoxylin-eosin staining, 100×) and Oil red-O staining (200×) and Triglyceride content in the liver. (**d**) Histological analysis of the liver (hematoxylin-eosin staining, 600×). The arrow heads denote ballooning necrosis of hepatocytes. Quantitative analysis of the number of hepatocytes showing ballooning necrosis. (**e**) The analysis of fibrosis by masson’s trichrome staining (400×). The arrow heads denote blue-colored pericellular fibrosis in the liver (upper panel 10×, lower panel 600×). Quantitative analysis of fibrotic area. Values are mean ± SEM. (**f**) Quantitative realtime PCR analysis of macrophage (*Cd68* and *F4/80*), inflammatory (*Tnfα*), pro-inflammatory cytokines (*Il-1β* and *Il-6*), and fibrotic markers (*Tgfβ*, *Procollagen 1a1, Collagen 3a1, and Collagen 4a1*). Data were compared by Student’s *t* test. (**g**) Immunohistochemical analysis using Ly-6C (a marker for neutrophils) and CD68 antibodies (n = 5 for each groups). The arrow heads denote Ly-6C- and CD68-potitive (a marker for macrophages or Kupffer cells) cells. (**f**) Quantitative analysis of the number of Ly-6C and CD68-positive cells in the liver. Data were compared by Student’s *t* test.

To test the beneficial effect of IGF-I on a NASH mouse model, we administered recombinant human IGF-I for 1 month by using osmotic pump on methionine-choline-deficient diet-fed *db/db* mice (MCD-*db*/*db*). The serum human IGF-I concentration was comparable to that of endogenous IGF-I concentration (Supplemental Fig. 1a,b). During IGF-I treatment, there were no significant changes in body weight as compared with the control group (Supplemental Fig. 2a). In the IGF-I treatment group, glucose tolerance and insulin sensitivity (Fig. 1a) were improved. In addition, the area of visceral adipose tissue was significantly decreased in the IGF-I treatment group (Fig. 1b). The concentrations of liver enzymes tended to decrease by IGF-I treatment (Table 1). Interestingly, the hepatic steatosis demonstrated by hematoxylin and eosin staining and Oil red-O staining (Fig. 1c) was markedly improved in the IGF-I treatment group. Tissue triglyceride content was significantly decreased by IGF-I treatment (Fig. 1c). Furthermore, the number of cells showing ballooning necrosis was decreased (Fig. 1d) and the fibrotic area visualized by Masson-trichrome staining was decreased (Fig. 1e) in the IGF-I treatment group compared with the control group. Quantitative analysis by realtime PCR (Fig. 1f) demonstrated that expression of the macrophage markers *Cd68* and *F4*/*80*, inflammation markers *Il-1β* and *Il*-*6*, and fibrotic markers *Procollagen 1a1* and *Collagen 4a1* were significantly decreased in the IGF-I treatment group. The immunohistochemical analysis using anti-Ly-6C and CD68 antibodies (Fig. 1g) showed a significant decrease in the number of positive cells in the IGF-I-treated group. These data clearly demonstrate the beneficial effect of IGF-I on steatosis, inflammation, and fibrosis in this NASH model.

### IGF-I improved mitochondrial function and oxidative stress, and inhibited hepatic stellate cell function

**Figure 2:**
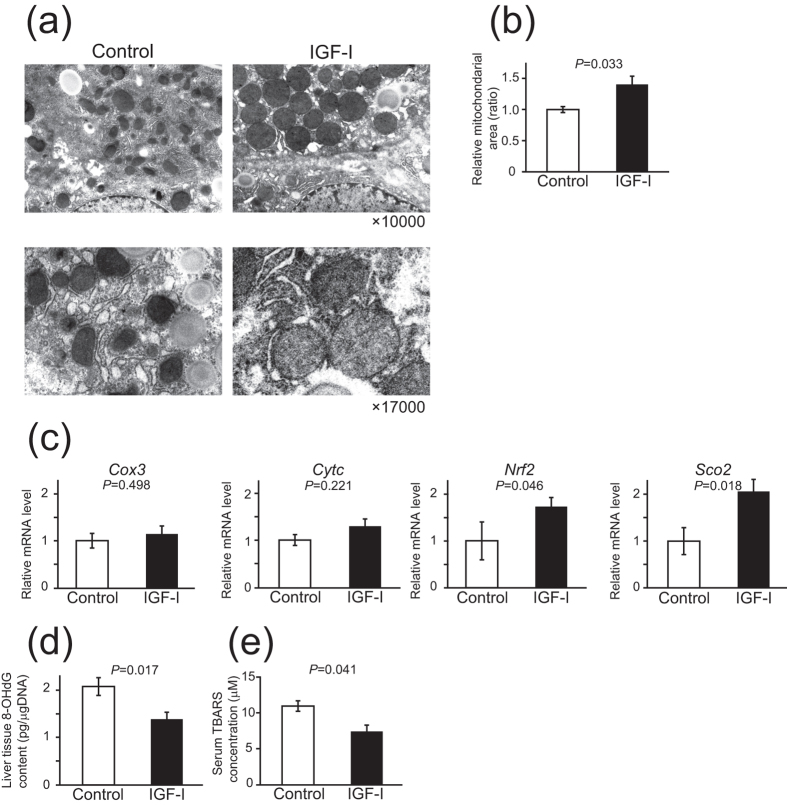
IGF-I improved the abnormalities in mitochondrial ultrastructure, mitochondrial gene expression, and oxidative stress markers in MCD-*db/db* NASH model mice. (**a**) Ultrastructure assessed by electron microscopy (original magnification 10,000×and 17,000×) of the liver tissue. In the control liver, the size of the mitochondria was smaller and heterogenous, shape was irregular, and the mitochondria showed profound cristae disorganization and irregular shapes. In contrast, IGF-I-treated mice showed restored mitochondrial structure. (**b**) Quantitative analysis of mitochondrial area showed that the area was significantly increased in the mice treated with IGF-I than in controls (n = 3 for each groups). (**c**) Quantitative realtime PCR analysis of mitochondrial functional genes (n = 5 for each groups). (**d,e**) Quantitative analysis of oxidative stress markers (n = 5 for each groups). (**d**) 8-OHdG levels in the liver tissue. (**e**) Serum TBARs levels. Data were compared by Student’s *t* test.

Because mitochondrial dysfunction and the related enhancement of oxidative stress play an important role in the development of NASH, we analyzed mitochondrial morphology by using electron microscopy and the expression of genes related to mitochondrial function. In liver of the NASH model, as shown in Fig. 2a for the control group, the morphology of the mitochondria was markedly deteriorated. The size was heterogeneous and the shape was deformed; however, in the IGF-I group, these changes were dramatically improved and the mitochondrial area was significantly increased (Fig. 2b). Among the genes related to mitochondrial function, the expression of *Nrf2* and *Sco2* was significantly increased in the IGF-I treatment group (Fig. 2c). Interestingly, the levels of marker for oxidative stress 8-OHdG in the liver and serum TBARS levels were significantly decreased in the IGF-I treatment group (Fig. 2d,e).

**Figure 3:**
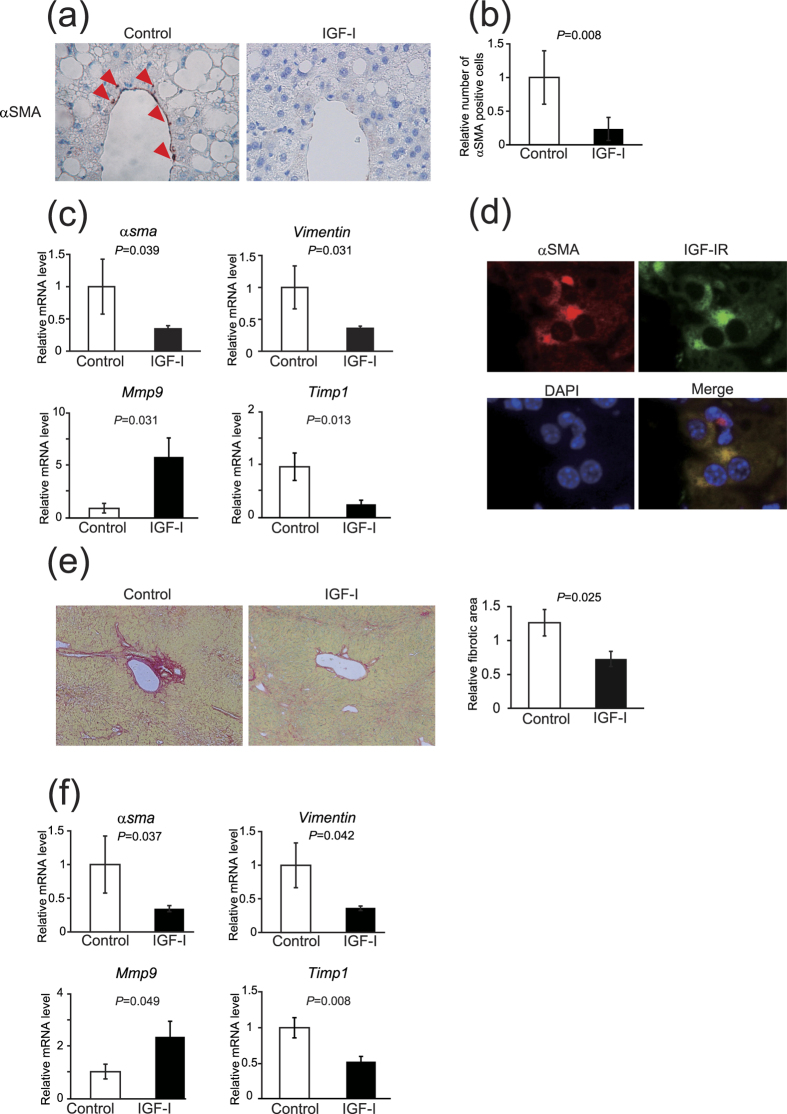
IGF-I treatment decreased the marker of activated HSCs in NASH model mice and improved hepatic fibrosis in cirrhosis model mice. (**a**) Immunohistological analysis of αSMA, which is an activated marker for HSCs in control or IGF-I-treated NASH model mice (n = 5 for each groups). (**b**) Quantitative analysis of αSMA-positive cells in the liver. (**c**) Quantitative realtime PCR analysis reveals decreased expression of HSCs marker (α*sma* and *Vimentin*) and *Timp-1* and increased expression of *Mmp9* in the IGF-I-treated liver. (**d**) Immunofluorescence analysis for double staining of αSMA and IGF-I receptor (IGF-IR) showed a co-localization in HSCs (800×). (**e**) The treatment with IGF-I in DMN-induced cirrhotic mice model (n = 5 for each groups). Human recombinant IGF-I or vehicle were administered cirrhotic mice using osmotic pump for 6 weeks. Sirius Red staining visualized the fibrosis of the liver (100×). Quantification of fibrosis based on Sirius Red staining. Fibrotic area was significantly decreased by IGF-I in DMN-induced cirrhotic mice. (**f**) Quantitative realtime PCR analysis revealed the decreased expression of *αsma*, *Vimentin,* and *Timp1* and increased expression of *Mmp9* by IGF-I treatment.

Because HSCs play a crucial role in the development of fibrosis, we analyzed the function of HSCs. Immunohistochemical analysis revealed a decrease in the number of αSMA-positive cells (Fig. 3a,b) by IGF-I treatment. Realtime PCR analysis comparably showed a decrease in the expression of activated HSCs markers (Fig. 3c). Increased *Mmp9* expression and decreased *Timp1* expression also indicated the inactivation of HSCs. In addition, immunohistochemical analysis clearly showed the expression of IGF-IR in the HSCs (Fig. 3d), suggesting a direct effect of IGF-I on HSCs.

### Anti-fibrotic effect of IGF-I in cirrhotic model mice

Next, we examined the effect of IGF-I on cirrhotic model mice treated with DMN. Similar to in the NASH model, IGF-I treatment clearly improved fibrosis (Fig. 3e) and decreased expression of activated HSCs markers, *αsma* and *Vimentin*, respectively (Fig. 3f), indicating that IGF-I ameliorated fibrosis in the cirrhotic model.

### IGF-I induced cellular senescence of HSCs *in vitro* and *in vivo*

**Figure 4:**
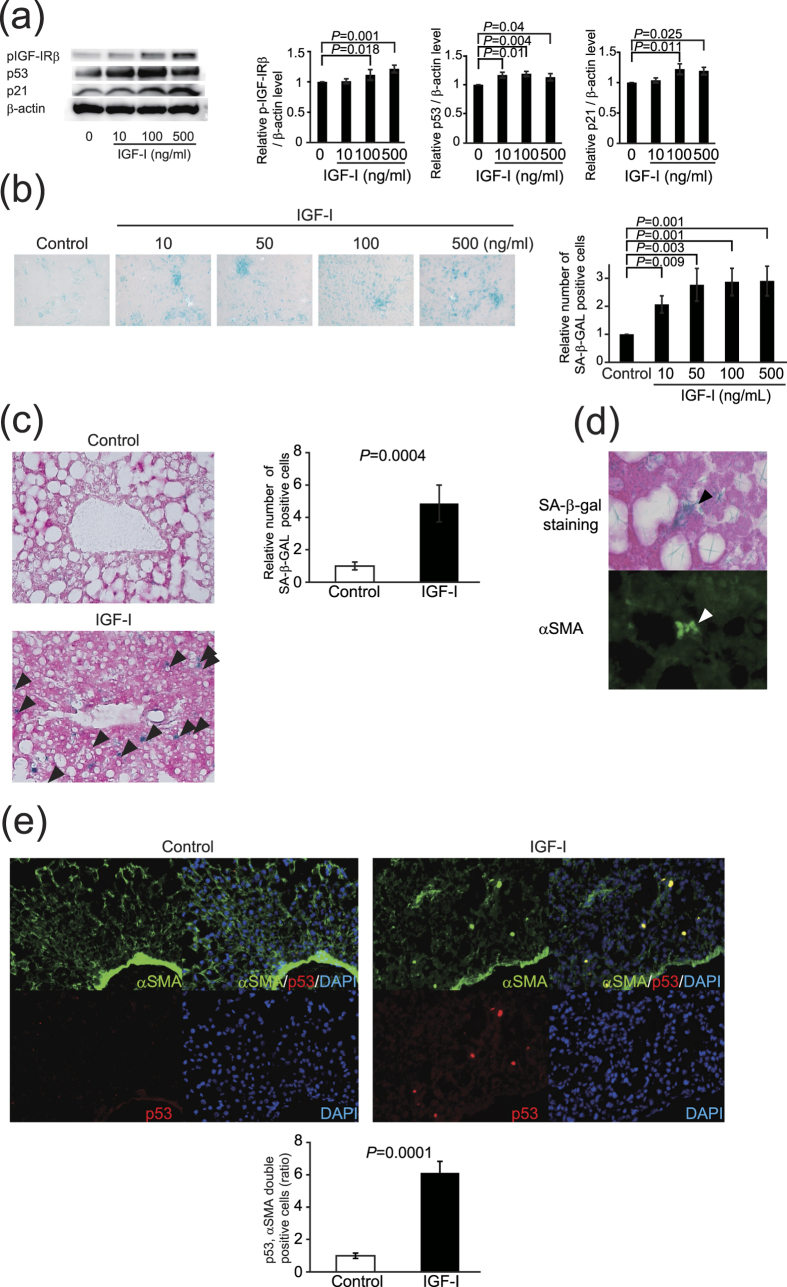
IGF-I induced cellular senescence of HSCs *in vitro* and *in vivo*. (**a**) IGF-I treatment increased cellular senescence markers p53 and p21 in rat primary HSCs demonstrated by immunoblotting. Cells were incubated with IGF-I for 72 h. Quantitative analysis of immunoblotting. Densitometric analyses were performed using data from 5 independent experiments. Each value was normalized to that of β-actin. Data were compared by Tukey’s honestly significant difference test. (**b**) IGF-I increased the number of SA-β-gal-positive cells in rat primary HSCs. HSCs were incubated with IGF-I at the indicated concentrations for 5 days (200×). Quantitative analysis of SA-β-gal-positive cells. Data are expressed as the mean ± SEM of 20 random fields (n = 10). (**c**) A presence of senescent cells in the liver evaluated by SA-β-gal assay (200×). The arrowheads denote senescent cells. There were increased senescent cells in IGF-I-treated NASH model mice. Quantification of senescence cells based on SA-β-gal staining. Data are expressed as the mean ± SEM of 20 random fields (n = 5). (**d**) Serial sections were evaluated by SA-β-gal staining and immunofluorescence for αSMA (800× SA-β-gal-positive cells were αSMA-positive, indicating that cellular senescence was induced in HSCs. (**e**) Double staining for αSMA and p53 showed the senescent HSCs (200×). The number of double-positive senescent HSCs was significantly increased by IGF-I treatment in NASH model mice. Data were compared by Student’s *t* test.

Recently, it was reported that cellular senescence of HSCs inhibits their function and limits fibrosis^4^; however, it is unknown which factor regulates cellular senescence in these cells. Because it has been reported that IGF-I induces cellular senescence in fibroblasts^24^, we hypothesized that IGF-I stimulates cellular senescence of HSCs. We then analyzed the effect of IGF-I on primary isolated rat HSCs. Intriguingly, the treatment with IGF-I significantly increased the protein levels of the senescence marker p21 and p53 in a dose-dependent manner (Fig. 4a). We further examined the senescence marker senescence-associated β-galactosidase (SA-β-gal) activity. As shown in Fig. 4b, treatment with IGF-I significantly increased the number of SA-β-gal-positive cells in HSCs. In addition, we confirmed the similar effect of IGF-I using LX2 cells, a human HSCs line^25^ (Supple Fig. 3a–d). These results indicate that IGF-I induces cellular senescence in HSCs *in vitro*. Furthermore, we examined the effect of IGF-I on cellular senescence *in vivo*. IGF-I treatment significantly increased SA-β-gal-positive cells in the NASH model mice (Fig. 4c). The staining of serial sections demonstrated that SA-β-gal-positive cells were αSMA-positive (Fig. 4d), indicating that cellular senescence was induced in the activated HSCs. In addition, the number of αSMA- and p53-double positive cells was increased by IGF-I treatment (Fig. 4e), clearly demonstrating that IGF-I induced cellular senescence of HSCs *in vivo*.

### p53 is necessary for IGF-I–induced cellular senescence and its anti-fibrotic effect

**Figure 5:**
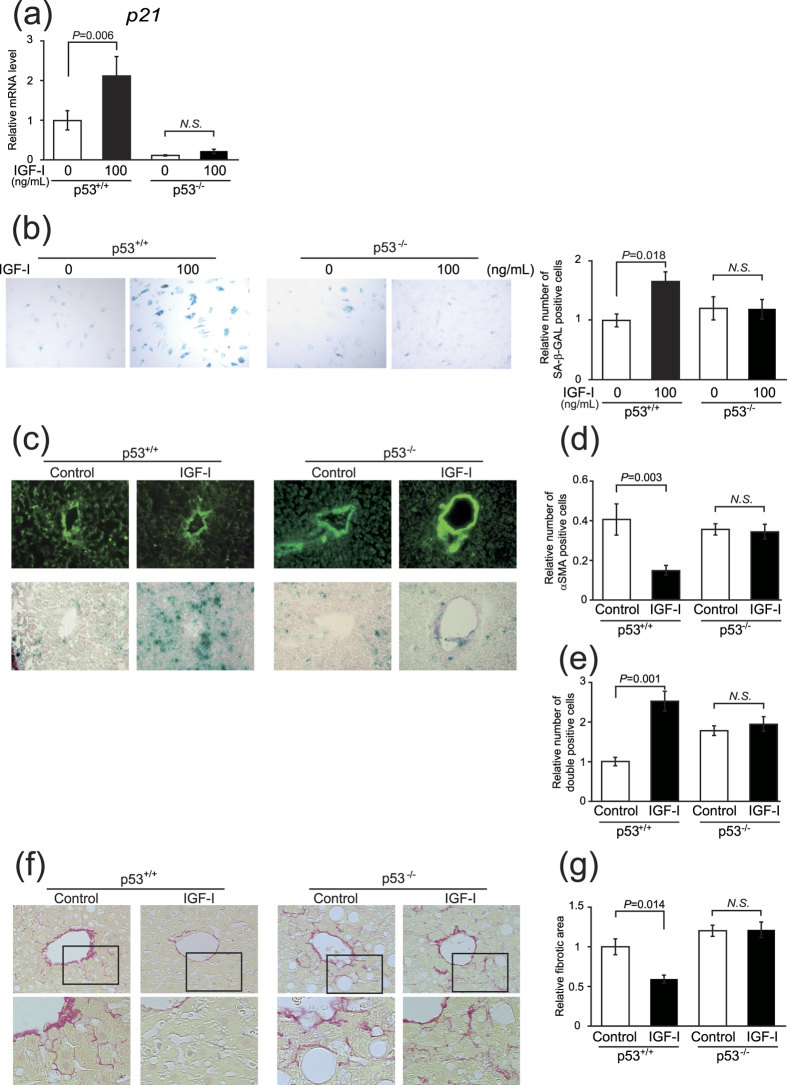
Intact senescence pathways were required for anti-fibrotic effect of IGF-I. (**a**) Quantitative realtime PCR analysis revealed the increased expression of *p21* in primary HSCs from p53^+/+^ mice by IGF-I treatment, but there was no change in the expression in those from p53^−/−^ mice. (**b**) Senescent primary HSCs visualized by SA-β-GAL assay (200×). Quantitative analysis of SA-β-GAL assay positive cells demonstrated that IGF-I increased senescent HSCs from p53^+/+^ mice; however, IGF-I showed no effect on HSCs from p53^−/−^ mice. Data were compared by Tukey’s honestly significant difference test. (**c**) MCD diet-fed-wild-type (WT) and -p53^−/−^ mice were treated with vehicle or IGF-I for 4 weeks (n = 5 for each groups). αSMA immunostaining (upper panel) and SA-β-gal staining (lower panel) on serial sections were performed (100×). (**d**) Quantification of αSMA-positive cells demonstrated that IGF-I decreased the number of αSMA-positive cells in WT mice; however, IGF-I did not decrease in p53^−/−^ mice. (**e**) Quantification of senescent HSCs, identified by double positive for SA-β-gal assay and αSMA immunostaining in the serial section as demonstrated in Fig. 4g. IGF-I increased the number of senescent HSCs cells in WT mice; however, IGF-I did not increase in p53^−/−^ mice. Data are expressed as the mean ± SEM of 20 random fields. (**f**) Fibrotic area visualized by Sirius Red staining (upper panel 200×, lower panel 400×). (**g**) Quantitative analysis of fibrotic area demonstrated that IGF-I decreased fibrotic area in WT mice; however, IGF-I showed any effect on p53^−/−^ mice. Data were compared by Tukey’s honestly test.

It is well-known that p53 plays a crucial role in cellular senescence^26^. We first examined the role of p53 in IGF-I induced senescence using primary HSCs from p53-deficient mice. Intriguingly, in contrast to the wild-type HSCs, IGF-I could not increase *p21* expression p53-deficient HSCs (Fig. 5a) and SA-β-GAL activity (Fig. 5b), clearly indicating that p53 is necessary for the IGF-I-induced senescence in HSCs. We next analyzed the effect of IGF-I on cellular senescence and anti-fibrotic effect using methionine-choline-deficient diet-fed p53-deficient mice. Because these mice were lean, the steatotic changes were mild; however, fibrotic changes were clearly observed. In wild-type mice, IGF-I significantly decreased the number of αSMA-positive cells; however, the number was unchanged by IGF-I treatment in p53-deficient mice (Fig. 5c,d). Furthermore, the analysis of serial sections by αSMA immunostaining and SA-β-gal activity revealed that while double-positive cells were increased by IGF-I treatment in wild-type mice, there was no effect of IGF-I in p53-deficient mice (Fig. 5e). Finally, we analyzed the effect of IGF-I on fibrosis using this mouse model. Intriguingly, treatment with IGF-I significantly ameliorated fibrotic changes in wild-type mice; however, IGF-I showed no effect in p53-deficient mice, indicating that the anti-fibrotic effect of IGF-I was p53-dependent (Fig. 5f,g).

## Discussion

We found that IGF-I ameliorated steatosis, and inflammation in the NASH animal model and exerted an anti-fibrotic effect on the NASH and cirrhotic animal models. IGF-I induced cellular senescence in HSCs, thereby inactivating these cells and preventing the progression of fibrosis in a p53-dependent manner. While the degree of fibrosis determines the prognosis in NASH and liver cirrhosis, efficacious treatment for fibrosis has not been established. We demonstrated that IGF-I has a potential therapeutic application for NASH and liver cirrhosis.

It has been reported that GH deficiency, in which hepatic IGF-I production is impaired in adults shows an increased prevalence of NASH in rats and humans^5^,^9^. GH or IGF-I treatment ameliorated NASH in an animal model, indicating that IGF-I plays a pivotal role in a GH-independent manner^9^. In clinical settings, serum IGF-I levels are decreased in patients with NAFLD^11^ and decreased levels of serum IGF-I are closely associated with the severity of fibrosis^12^. These data also suggest that impaired IGF-I secretion even in the physiological range may be causally related to the development of fibrosis in NAFLD. In agreement with this, we showed that IGF-I administration targeting a physiological serum level ameliorated the fibrosis. The liver is the main source of serum IGF-I^7^ and secreted IGF-I considered to exert its action on various organs in an endocrine fashion. The present data suggest that IGF-I produced by hepatocytes locally acts on HSCs and regulates their function in a paracrine manner.

Several underlying mechanisms of IGF-I action in the liver have been suggested. IGF-I ameliorates liver damage in rats with GH deficiency concomitant with the improvement in oxidative stress and mitochondrial function, which are important factors in the development of NASH. Multiple methods of delivery of IGF-I to the liver in the cirrhotic animal models have been shown to improve fibrosis^15^,^21^,^22^. Upregulation of hepatocyte growth factor and downregulation of transforming growth factor β-1 were observed following IGF-I overexpression^22^. In the present study, we clearly demonstrated that IGF-I induced cellular senescence in HSCs both *in vitro* and *in vivo*. In mice lacking the key senescence regulator p53, IGF-I did not induce cellular senescence in HSCs and show any effect on fibrosis. These results demonstrated a novel mechanism for the anti-fibrotic action of IGF-I in HSCs.

The underlying mechanism that IGF-I induces senescence in HSCs is unknown. Although we have clearly shown that p53 is necessary for the IGF-I-induced senescence, it has not been fully elucidated how IGF-I induces p53 expression. It has been reported that IGF-I induces cellular senescence in murine and human fibroblast via oxidative stress by activating NADPH oxidase^24^,^27^, suggesting that IGF-I stimulates intracellular ROS production in HSCs, increases p53 expression, and induces senescence in HSCs.

Recently, it has been reported that p53 play a role in not only HSCs but also in hepatocytes in the development of NASH^28^. In the present study, we used mice that were deficient in p53 in whole body, indicating that we cannot exclude the indirect influence of p53 deficiency in the other cells or tissues including hepatocytes. However, the results of impaired induction in senescence in primary HSCs from p53^−/−^ mice by IGF-I strongly support a presence of p53-dependent direct action of IGF-I in HSCs.

All patients with NAFLD require advice for life style modifications aimed at weight loss and increased physical activity, as well as the treatment of any associated risk factors^29^. It has been reported that some pharmacotherapies directed at the liver are effective for treating NASH. A recent meta-analysis demonstrated that pioglitazone treatment significantly improved steatosis, inflammation, and to a lesser degree, fibrosis^30^. Treatment with the antioxidant vitamin E improved steatosis and inflammation but not fibrosis^31^. Thus far, efficacious therapy for fibrosis has not been established. In this regard, IGF-I treatment is a potential therapeutic option particularly for fibrosis via a different mechanism.

A primary concern of clinical application of IGF-I is an increased risk of cancer. According to the present data, a physiological, and not pharmacological, range of IGF-I improved fibrosis; however, even in the physiological range, increased serum IGF-I levels were associated with an increased risk of prostate, breast, and colon cancer^32^. The results of cellular and animal models suggest that the IGF-I signaling pathway plays an important role in the development of hepatocellular carcinoma and clinical trials targeting the IGF-I axis with a focus on the anti-IGF-IR approach have been conducted^33^. Careful observation for the development of hepatocellular carcinoma is necessary, particularly when IGF-I is used for cirrhosis. Nevertheless, prevention of fibrosis is an essential target for the treatment of NASH and cirrhosis^34^, suggesting a beneficial effect of IGF-I treatment on these conditions.

In conclusion, we demonstrated that IGF-I induces senescence of HSCs, inactivates these cells and limits fibrosis in a p53-dependent manner and a potential therapeutic application of IGF-I for the treatment of NASH and cirrhosis.

## Methods

### Animal model and experimental design

Eight-week-old male ICR mice, Sprague-Dawley (SD) rats, and *db*/*db* mice with a C57BL/6 background were purchased from CLEA Japan, Inc. (Tokyo, Japan). The p53^−/−^ mouse strain (RBRC01361) was provided by RIKEN BRC (Tsukuba, Japan) through the National Bio-Resource Project of the MEXT, Japan^35^. Mice and rats were maintained in a temperature-controlled and light-controlled facility (12-h light, 12-h dark), and permitted *ad libitum* consumption of water. Mice were fed methionine-choline-deficient diet (Oriental Yeast Co., Tokyo, Japan) for 6 weeks to establish the NASH model. Dimethylnitrosamine (DMN) was injected intraperitoneally in doses of 10 μg per 1 g body weight (diluted 1:2000 with 0.15 M sterile NaCl). The injections were given on three consecutive days of each week for a period of 9 weeks. DMN-treated animals were sacrificed on days 70 from the beginning of exposure. Mice were anesthetized using isoflurane (Dainippon Sumitomo Pharma Co., Ltd, Osaka, Japan) and nembutal (Abbott Laboratories, Abbott Park, IL,) and osmotic minipumps (model 2004D, Alzet Co., Palo Alto, CA), containing porcine GH (10 mg/mL; NIDDK National Hormone and Pituitary Program) or recombinant human IGF-I (10 mg/mL; Astellas Pharma, Co., Ltd., Tokyo, Japan), vehicle (saline) were implanted subcutaneously. Four weeks (NASH model) or 6 weeks (cirrhosis model) after pump-placement, the mice were sacrificed; a portion of each liver was fixed in 4% paraformaldehyde phosphate buffer for histology. Additional samples were snap-frozen in liquid nitrogen for later analysis of liver lipid levels and lipid peroxidation. This study was approved by the Institutional Animal Care and Use Committee (Permission number: P120904, P150203) and carried out according to the Kobe University Animal Experimentation Regulation.

### Computed tomography

Twelve-week-old mice were anesthetized with Isoflurane. The percent body fat and visceral fat mass ratio of mice were analyzed using Latheta LTC-100 (ALOKA, Tokyo, Japan).

### Cell culture and treatments

LX-2, an immortalized human HSC line^25^, was cultured in Dulbecco’s modified eagle medium (DMEM) containing 2% FBS. The medium was then replaced with serum-free-DMEM containing penicillin/streptomycin and glutamine for 24 h before experimentation. Primary HSCs were separated from SD rat livers and p53-deficient mice in an inverted gradient as described previously^36^.

### Measurement of plasma and liver biochemical parameters

To quantify hepatic triglyceride content, the liver was lysed with buffer from a commercially available kit (TG E-test; Wako, Osaka, Japan) and disrupted by sonication according to the manufacturer’s instructions^37^. Tissue 8-OHdG levels were determined using DNA Extractor(R) TIS Kit (catalog no. 296-67701, Wako), 8-OHdG Assay Preparation Reagent Set (catalog no. 292-67801, Wako), and High Sensitive 8-OHdG Check ELISA kit (KOG-HS10E, catalog no. 307-07921, NIKKEN SEIL Co., Ltd, Sizuoka, Japan). Blood samples were centrifuged, and serum or plasma was frozen at −80 °C for subsequent measurement of aspartate aminotransferase (AST), alanine aminotransferase (ALT), free fatty acid (FFA), triglyceride, insulin, IGF-I, and thiobarbituric1 acid-reactive substances (TBARs) levels. AST and ALT levels were measured using the transaminase C-II test kit (Wako). FFA and triglyceride levels were measured using the nonesterified fatty acid C-test (Wako) and triglyceride E-test (Wako), respectively. Mouse insulin levels were measured using the Mouse Insulin ELISA kit (Shibayagi, Gunma, Japan). Human total IGF-I (Quantikine Human IGF-I Immunoassay, catalog no. DG100, R&D systems, Inc., Minneapolis, MN, USA) and mouse/rat total IGF-I (Diagnostic Systems Laboratories, Inc., Webster, TX, USA) levels were measured using an ELISA or EIA kit according to the manufacturer’s instructions. Serum TBARs level was measured using the TBARS assay kit (Cayman Chemical Company, Ann Arbor, MI, USA).

### Western blotting analysis

Expressions of phospho-IGF-I receptor (1:1000, Cell Signaling Technology, Inc.), αSMA (1:500, Dako, Denmark), p53 (1:1000, Cell Signaling Technology, Inc., USA), p21 (1:1000, Santa Cruz Biotechnology, Inc., Santa Cruz, CA, USA), and β actin (1:2000, Sigma-Aldrich Co., St. Louis, MO, USA) were determined using SDS-polyacrylamide gel electrophoresis (SDS-PAGE) and western blotting analysis. Membrane-bound antibodies were visualized using horse-radish peroxidase-conjugated secondary antibodies and subsequent ECL detection (Immunostar LD, Wako).

### RNA extraction and Quantitative real time PCR

Total RNA was extracted from the liver tissue using Trizol reagent (Invitrogen). Five-hundred ng of RNA was reverse transcribed into cDNA by reverse transcription with ReverTra Ace qPCR RT kit (TOYOBO Co., Ltd., Osaka, Japan). Real-time quantitative PCR was performed using the ABI Prism 7500 Sequence Detection System (Applied Biosystems, Foster City, CA). PCR was catalyzed by SYBR^®^ Premix Ex Taq™ II (TAKARA BIO Inc, Shiga, Japan), while SYBR Green I DNA-binding dye generated the fluorescence signals during each of the 40 cycles, in proportion to the quantities of double-stranded DNA. The expression levels of all genes were normalized to 18S or GAPDH gene expression level. Each experiment was conducted in duplicate and the average was calculated. The sequence of primers used was shown in Supplemental Table 1.

### Senescence-associated β-galactosidase staining

Detection of Senescence-associated β-galactosidase (SA-β-gal) activity was performed at pH = 5.5 for mouse/rat tissue and pH = 6.0 for human cells as described previously^38^. To avoid staining due to cell confluence rather than proliferative senescence^39^, the assay was performed in sub-confluent cultures displaying comparable cell densities.

### Histological analysis

For routine histology, liver specimens were preserved in 4% paraformaldehyde in PBS and dehydrated in a graded alcohol series. Following xylene treatment, the specimens were embedded in paraffin blocks and cut into 5-μm thick sections, which were stained with hematoxylin-eosin for routine examination or with Masson’s trichrome and Sirius Red, and Oil red-O staining. A hepatopathologist who was blinded to the experimental conditions examined all sections for steatosis, inflammation, and fibrosis. To assess hepatic steatosis, the histopathological criteria proposed by Brunt *et al.* was adopted^40^,^41^,^42^,^43^. The degree of fibrosis was assessed by digital morphometry. The number of pixels corresponding to fibrosis was measured as a percentage of the total pixels of each image by using NIH Image J software (http://rsb.info.nih.gov/ij/). Immunostaining was performed as previously described^44^. The following antibodies were used: Ly-6C (1:200, Santa cruz Biotechnology, Inc. Texas, USA.), CD68 (1:100, Bioss Inc., Boston, USA), αSMA (1:500, Dako, Glostrup, Denmark). Biotinylated anti-goat immunoglobulins (LSAB + System-HRP, Dako) and Peroxidase-conjugated immunepolymer for rabbit polyclonal antibodies (Envision-PO for rabbit, Dako) were used for signal detection as secondary antibody. AlexaFluor-conjugated secondary antibodies were used for immunofluorescent signal detection. All fluorescence microscopy was performed in confocal mode (LSM700, Zeiss, Jena, Germany and BZ8100, Keyence, Osaka, Japan).

### Transmission electron microscopy

Liver tissue was cut into small sections, fixed by 1% OsO_4_ fixative solution and observed using a Jeol JEM-1230 transmission electron microscope (Tokyo, Japan) equipped with a Gatan bioscan camera mode l792 and TEM AutoTune™ (Nippon Gatan, Tokyo, Japan).

### Statistical analyses

Values were expressed as the mean ± SEM. Significance was established using Student’s *t* test or analysis of variance as appropriate. Results were considered to be statistically significant at values of *p* < 0.05.

## Additional Information

**How to cite this article**: Nishizawa, H. *et al.* IGF-I induces senescence of hepatic stellate cells and limits fibrosis in a p53-dependent manner. *Sci. Rep.* **6**, 34605; doi: 10.1038/srep34605 (2016).

## Supplementary Material

Supplementary Information

## Figures and Tables

**Table 1:** Biochemical data in the control and IGF-I-treated group of MCD-*db/db* mice after 4 weeks of treatment.

	Control	IGF-I	*P* value
Body weight (g)	37.6 ± 1.5	37.3 ± 0.9	0.87
AST (IU/L)	337.5 ± 43.1	317.3 ± 48.4	0.72
ALT (IU/L)	173.4 ± 34.1	147.0 ± 20.7	0.41
FFA (mEq/L)	0.45 ± 0.03	0.62 ± 0.07	0.78
Triglyceride (mg/dL)	59.6 ± 7.5	71.5 ± 4.9	0.54

Data were compared by Student’s *t* test.

Values are mean ± SEM.
