# Supplementary material for: IGF-I induces senescence of hepatic stellate cells and limits fibrosis in a p53-dependent manner
Source: Sci Rep. 2016 Oct 10;6:34605. doi: 10.1038/srep34605 (PMC5056388; doi:10.1038/srep34605)
Supplement: Supplementary Information [file srep34605-s1.pdf]

IGF-I induces senescence of hepatic stellate cells and limits fibrosis in a p53-dependent manner.

Hitoshi Nishizawa[2], Genzo Iguchi[2], Hidenori Fukuoka[2], Michiko Takahashi[3], Kentaro Suda[1], Hironori Bando[1], Ryusaku Matsumoto[1], Kenichi Yoshida[1], Yukiko Odake[1], Wataru Ogawa[1], Yutaka Takahashi[1*]

[1]Division of Diabetes and Endocrinology, Department of Internal Medicine, Kobe University Graduate School of Medicine, Kobe, Japan

[2]Division of Diabetes and Endocrinology, Kobe University Hospital, Japan [3]Department of Nutrition, Kobe University Hospital, Japan

Supplementary Table1

RT-PCR Primers for Analysis

Gene	Organism	Direction	Sequence
Cd68	mouse	Forward	GACACTTCGGGCCATGTT
		Reverse	GAGGAGGACCAGGCCAAT
F4/80	mouse	Forward	GGAGGACTTCTCCAAGCCTATT
		Reverse	AGGCCTCTCAGACTTCTGCTT
Tnfα	mouse	Forward	CCGATGGGTTGTACCTTGTC
		Reverse	CGGACTCCGCAAAGTCTAAG
Tgfβ	mouse	Forward	TTGCCCTCTACAACCAACACAA
		Reverse	GGCTTGCGACCCACGTAGTA
Il-1β	mouse	Forward	TTGACGGACCCCAAAAGAT
		Reverse	GAAGCTGGATGCTCTCATCTG
Il-6	mouse	Forward	GCTACCAAACTGGATATAATCAGGA
		Reverse	CCAGGTAGCTATGGTACTCCAGAA
Procollagen1a1	mouse	Forward	GAGCGGAGAGTACTGGATCG
		Reverse	GTTCGGGCTGATGTACCAGT
Collagen3a1	mouse	Forward	TCCCCTGGAATCTGTGAATC
		Reverse	TGAGTCGAATTGGGGAGAAT
Collagen4a1	mouse	Forward	TTAAAGGACTCCAGGGACCAC
		Reverse	CCCACTGAGCCTGTCACAC
Mmp3	mouse	Forward	TTGTTCTTTGATGCAGTCAGC
		Reverse	GATTTGCGCCAAAAGTGC
Mmp9	mouse	Forward	ACGACATAGACGGCATCCA
		Reverse	GCTGTGGTTCAGTTGTGGTG
Timp1	mouse	Forward	ATTCAAGGCTGTGGGAAATG
		Reverse	CTCAGAGTACGCCAGGGAAC
Cox3	mouse	Forward	GCAGGATTCTTCTGAGCGTTCT
		Reverse	GTCAGCAGCCTCCTAGATCATGT
Cyt c	mouse	Forward	GCAAGCATAAGACTGGACCAAA
		Reverse	TTGTTGGCATCTGTGTAAGAGAATC
Nrf2	mouse	Forward	CCAGAAGCCACACTGACAGA
		Reverse	GGAGAGGATGCTGCTGAAAG
Sco2	mouse	Forward	ATCGCACAGCCCTAAGTCTC
		Reverse	CAGTAGCATCGTGGACCTGA
α*sma*	mouse	Forward	AAACAGGAATACGACGAAG
		Reverse	CAGGAATGATTTGGAAAGGA
Vimentin	mouse	Forward	CCAACCTTTTCTTCCCTGAA
		Reverse	TGAGTGGGTGTCAACCAGAG
Fibronectin	mouse	Forward	CGGAGAGAGTGCCCCTACTA
		Reverse	CGATATTGGTGAATCGCAGA
p21	mouse	Forward	AACATCTCAGGGCCGAAA
		Reverse	TGCGCTTGGAGTGATAGAAA
18s	mouse	Forward	TCAAGAACGAAAGTCGGAGG
		Reverse	GGACATCTAAGGGCATCACA
Gapdh	mouse	Forward	AGCTTGTCATCAACGGGAAG
		Reverse	TTTGATGTTAGTGGGGTCTCG
α*sma*	rat	Forward	TCCCAAGCTGTGTGTCTCTG
		Reverse	GTGCCACGTTATGATGATGC
Vimentin	rat	Forward	CTCACCTGCGAAGTGGATG
		Reverse	TCTCTTCCATTTCACGCATCT
Igf-1	mouse	Forward	GCTTGCTCACCTTCACCAGC
		Reverse	AATGTACTTCCTTCTGAGTCT

Supplementary Table 1 Primer sequence for realtime PCR analysis

Supplementary Figure 1

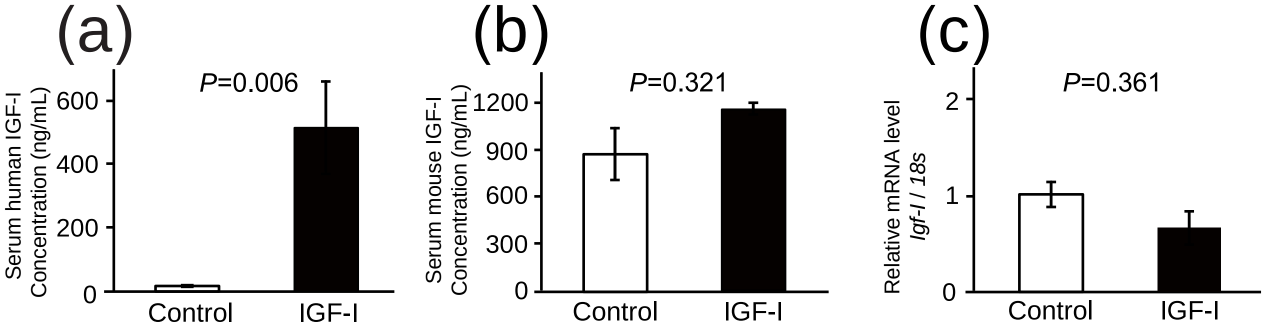

Supplementary Figure 1 (a) Serum human IGF-I concentrations measured using human IGF-I-specific ELISA kit in NASH model mice. (b) Serum mouse IGF-I concentrations measured using mouse IGF-I-specific ELISA kit. (c) Quantitative realtime PCR analysis revealed no changes in mRNA expression of endogenous *Igf-I* in the liver. Data were compared by Student's *t* test.

Supplementary Figure 2

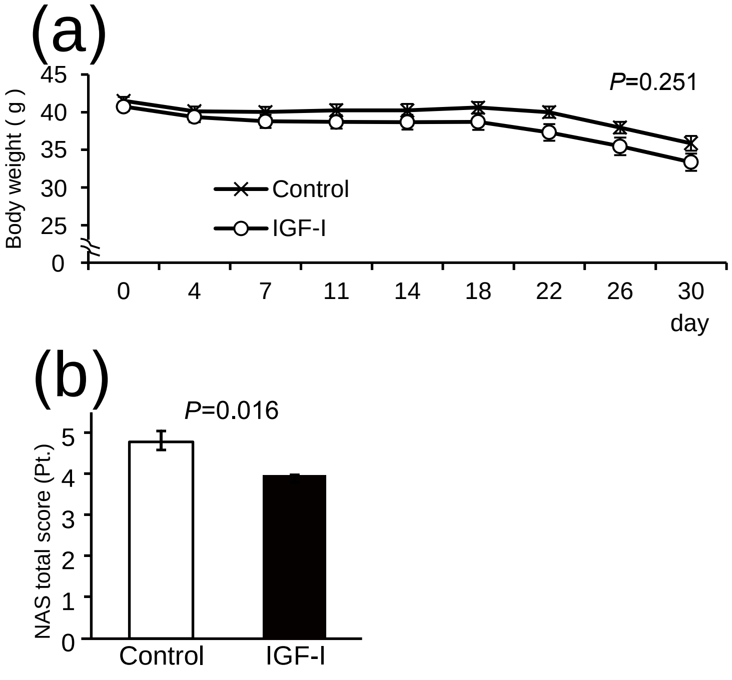

Supplementary Figure 2 (a) Changes in the body weight during IGF-I treatment in the NASH model mice. Data were compared by MANOVA test. (b) Total NAFLD activity score (NAS) was evaluated by a blinded pathologists. NAS was assessed on a scale of 0-8, with higher scores indicating more severe disease; the components of this measure include steatosis (assessed on a scale of 0-3), lobular inflammation (assessed on a scale of 0-3), and hepatocellular ballooning (assessed on a scale of 0-3). SData were compared by tudent's *t* test, $p < 0.05$.

Supplementary Figure 3

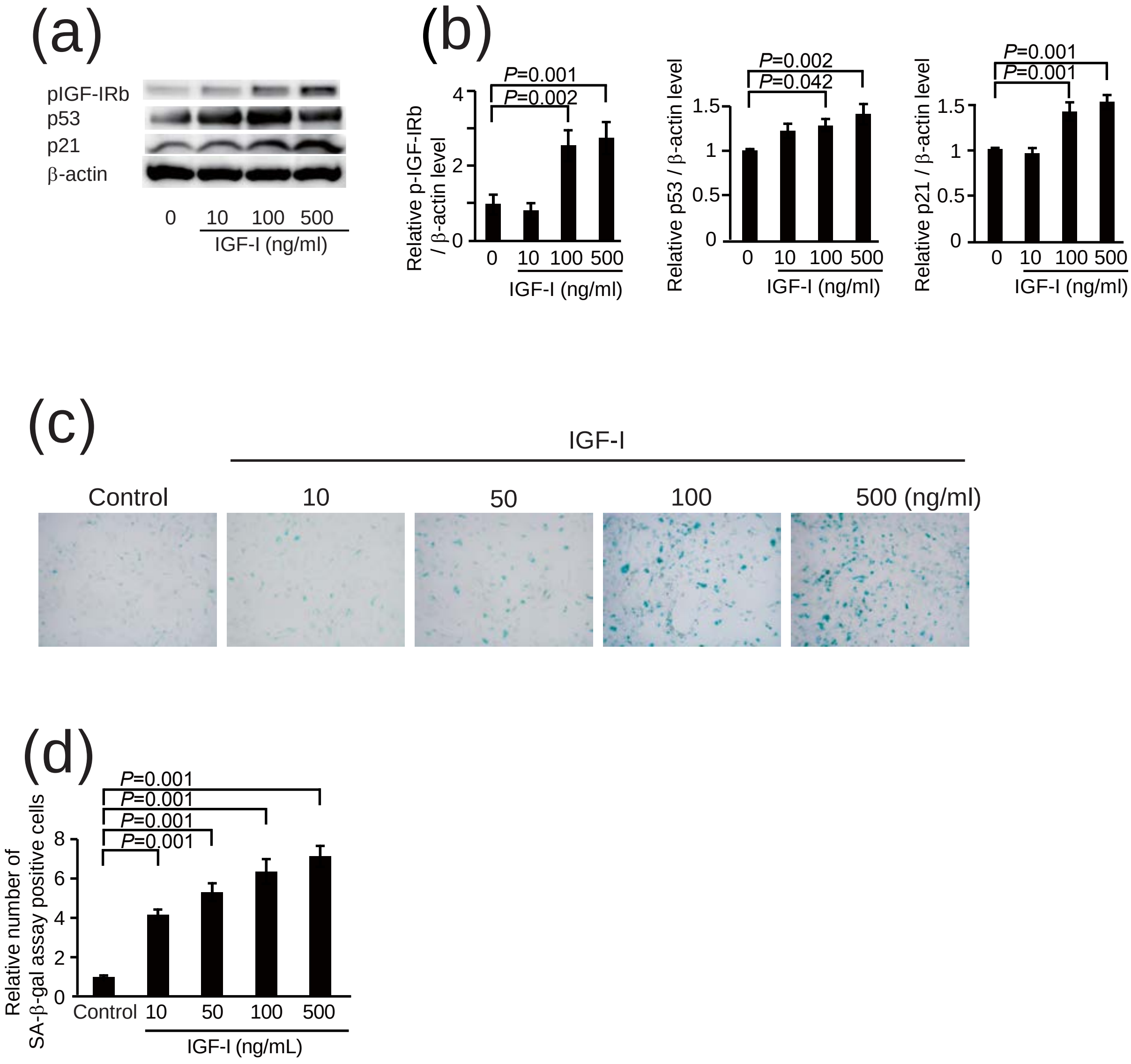

Supplementary Figure 3 IGF-I induced cellular senescence in human hepatic stellate cell line, LX2 cells.

(a) IGF-I increased expressions of p53 and p21 proteins in a concentration-dependent manner. Cells were incubated with IGF-I for 72 h. (b) Quantitative analysis demonstrated that IGF-I significantly increased p53 and p21 protein expression and phosphorylation of the IGF-I receptor in a concentration-dependent manner. Densitometric analyses were performed using data from 5 independent experiments. Each value was normalized to that of β-actin. (c) IGF-I induced SA-β-gal activation in LX2 cells. HSCs were incubated with IGF-I at the indicated concentrations for 5 days. (SA-β-gal staining, 200×). (d) Quantitative analysis demonstrated that IGF-I significantly increased SA-β-gal-positive cells in a concentration-dependent manner. Data were expressed as the mean ± SEM of 20 random fields (n = 10). Data were compared by Tukey's honestly significant difference test.
